# Dibromido{2-morpholino-*N*-[1-(2-pyrid­yl)ethyl­idene]ethanamine-κ^3^ *N*,*N*′,*N*′′}zinc(II)

**DOI:** 10.1107/S1600536811002753

**Published:** 2011-01-26

**Authors:** Yan-Wei Ding, Xiao-Ling Wang, Li-Li Ni

**Affiliations:** a College of Chemistry and Chemical Engineering, Liaoning Normal University, Dalian 116029, People’s Republic of China; b The Miyun High School Attached to Capital Normal University, Beijing 101500, People’s Republic of China

## Abstract

In the title complex, [ZnBr_2_(C_13_H_19_N_3_O)], the Zn^II^ atom is five-coordinated by the three N-donor atoms of the Schiff base ligand and by two Br atoms in a distorted square-pyramidal geometry. The morpholine ring adopts a chair conformation.

## Related literature

For background to Schiff base complexes, see: Dhar & Chakravarty (2003 ▶); Das *et al.* (2006 ▶); Nayak *et al.* (2006 ▶). For the crystal structures of similar Schiff base–zinc(II) complexes, see: Wang (2010 ▶); Zhu *et al.* (2007 ▶); Li & Zhang (2004 ▶); Zhu & Yang (2008 ▶).
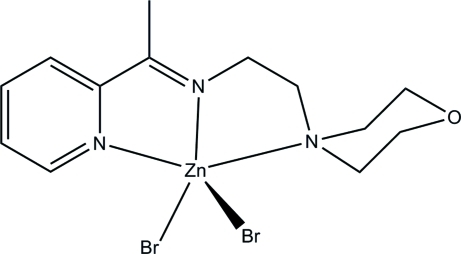


## Experimental

### 

#### Crystal data

[ZnBr_2_(C_13_H_19_N_3_O)]
*M* *_r_* = 458.50
Monoclinic, 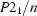

*a* = 9.831 (4) Å
*b* = 13.985 (6) Å
*c* = 12.183 (5) Å
β = 106.626 (4)°
*V* = 1604.9 (11) Å^3^
*Z* = 4
Mo *K*α radiation
μ = 6.51 mm^−1^
*T* = 298 K
0.35 × 0.32 × 0.32 mm

#### Data collection

Bruker SMART CCD area-detector diffractometer
Absorption correction: multi-scan (*SADABS*; Bruker, 2001 ▶) *T*_min_ = 0.209, *T*_max_ = 0.230
12426 measured reflections
3411 independent reflections
2159 reflections with *I* > 2σ(*I*)
*R*_int_ = 0.108

#### Refinement

*R*[*F* ^2^ > 2σ(*F* ^2^)] = 0.065
*wR*(*F* ^2^) = 0.177
*S* = 1.05
3411 reflections
182 parameters
H-atom parameters constrained
Δρ_max_ = 1.35 e Å^−3^
Δρ_min_ = −1.26 e Å^−3^

### 

Data collection: *SMART* (Bruker, 2007 ▶); cell refinement: *SAINT* (Bruker, 2007 ▶); data reduction: *SAINT*; program(s) used to solve structure: *SHELXS97* (Sheldrick, 2008 ▶); program(s) used to refine structure: *SHELXL97* (Sheldrick, 2008 ▶); molecular graphics: *SHELXTL* (Sheldrick, 2008 ▶); software used to prepare material for publication: *SHELXTL* and *publCIF* (Westrip, 2010 ▶).

## Supplementary Material

Crystal structure: contains datablocks global, I. DOI: 10.1107/S1600536811002753/su2250sup1.cif

Structure factors: contains datablocks I. DOI: 10.1107/S1600536811002753/su2250Isup2.hkl

Additional supplementary materials:  crystallographic information; 3D view; checkCIF report

## Figures and Tables

**Fig. 1.**
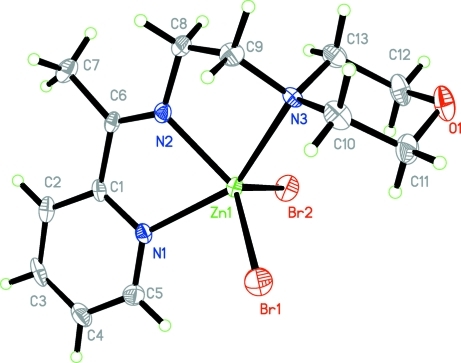
Molecular structure of the title compound, with displacement ellipsoids drawn at the 30% probability level.

## supplementary crystallographic information

### Comment

In the last few years, considerable attention has focused on the preparation and properties of Schiff base complexes (Dhar & Chakravarty, 2003; Das *et al.*, 2006; Nayak *et al.*, 2006). Herein we report on the crystal structure of a new Schiff base zinc(II) complex.

The molecular structure of the title mononuclear zinc(II) complex is shown in Fig. 1. The Zn atom is five-coordinated by the three N-donor atoms of the Schiff base ligand, and by two Br atoms in a distorted square-pyramidal geometry. All the coordinate bond lengths are within normal values and are comparable to those in similar zinc(II) complexes with Schiff bases (Wang, 2010; Zhu *et al.*, 2007; Li & Zhang, 2004; Zhu & Yang, 2008). As expected, the morpholine ring adopts a chair conformation.

### Experimental

The title complex was prepared by the reaction of 2-acetylpyridine (0.20 g, 1.65 mmol), 4-(2-aminoethyl)morpholine (0.21 g, 1.65 mmol), and zinc bromide (0.37 g, 1.65 mmol) in methanol at ambient temperature. Colourless block-like single crytals were formed by slow evaporation of the solution in air.

### Refinement

The C-bound H-atoms were included in calculated positions and treated as riding atoms: C-H = 0.93, 0.97 and 0.96 Å for CH, CH_2_ and CH_3_ H-atoms, respectively, with U_iso_(H) = k × U_eq_(C), where k = 1.2 for CH and CH_2_ H-atoms, and 1.5 for CH_3_ H-atoms. The highest residual density peak, 1.35 e Å^-2^, is 0.58 Å from atom Zn1, while the deepest residual density hole, -1.26 e Å^-2^, is 0.62 Å from atom Br1.

### Crystal data

**Table:**

[ZnBr_2_(C_13_H_19_N_3_O)]	*F*(000) = 904
*M**_r_* = 458.50	*D*_x_ = 1.898 Mg m^−^^3^
Monoclinic, *P*2_1_/*n*	Mo *K*α radiation, λ = 0.71073 Å
Hall symbol: -P 2yn	Cell parameters from 1887 reflections
*a* = 9.831 (4) Å	θ = 2.3–25.1°
*b* = 13.985 (6) Å	µ = 6.51 mm^−^^1^
*c* = 12.183 (5) Å	*T* = 298 K
β = 106.626 (4)°	Block, colourless
*V* = 1604.9 (11) Å^3^	0.35 × 0.32 × 0.32 mm
*Z* = 4	

### Data collection

**Table:**

Bruker SMART CCD area-detector diffractometer	3411 independent reflections
Radiation source: fine-focus sealed tube	2159 reflections with *I* > 2σ(*I*)
graphite	*R*_int_ = 0.108
ω scans	θ_max_ = 27.0°, θ_min_ = 2.3°
Absorption correction: multi-scan (*SADABS*; Bruker, 2001)	*h* = −12→12
*T*_min_ = 0.209, *T*_max_ = 0.230	*k* = −17→17
12426 measured reflections	*l* = −15→15

### Refinement

**Table:**

Refinement on *F*^2^	Primary atom site location: structure-invariant direct methods
Least-squares matrix: full	Secondary atom site location: difference Fourier map
*R*[*F*^2^ > 2σ(*F*^2^)] = 0.065	Hydrogen site location: inferred from neighbouring sites
*wR*(*F*^2^) = 0.177	H-atom parameters constrained
*S* = 1.05	*w* = 1/[σ^2^(*F*_o_^2^) + (0.0584*P*)^2^ + 5.9893*P*] where *P* = (*F*_o_^2^ + 2*F*_c_^2^)/3
3411 reflections	(Δ/σ)_max_ < 0.001
182 parameters	Δρ_max_ = 1.35 e Å^−^^3^
0 restraints	Δρ_min_ = −1.26 e Å^−^^3^

### Special details

**Table:**

Geometry. All e.s.d.'s (except the e.s.d. in the dihedral angle between two l.s. planes) are estimated using the full covariance matrix. The cell e.s.d.'s are taken into account individually in the estimation of e.s.d.'s in distances, angles and torsion angles; correlations between e.s.d.'s in cell parameters are only used when they are defined by crystal symmetry. An approximate (isotropic) treatment of cell e.s.d.'s is used for estimating e.s.d.'s involving l.s. planes.
Refinement. Refinement of *F*^2^ against ALL reflections. The weighted *R*-factor *wR* and goodness of fit *S* are based on *F*^2^, conventional *R*-factors *R* are based on *F*, with *F* set to zero for negative *F*^2^. The threshold expression of *F*^2^ > σ(*F*^2^) is used only for calculating *R*-factors(gt) *etc*. and is not relevant to the choice of reflections for refinement. *R*-factors based on *F*^2^ are statistically about twice as large as those based on *F*, and *R*- factors based on ALL data will be even larger.

### Fractional atomic coordinates and isotropic or equivalent isotropic displacement parameters (Å^2^)

**Table:**

	*x*	*y*	*z*	*U*_iso_*/*U*_eq_	
Zn1	0.27265 (9)	0.91109 (6)	0.83908 (8)	0.0207 (3)	
Br1	0.45896 (10)	0.79761 (8)	0.87089 (9)	0.0448 (3)	
Br2	0.04314 (10)	0.86043 (7)	0.72941 (9)	0.0418 (3)	
O1	0.3163 (7)	0.9365 (6)	0.4853 (6)	0.0490 (19)	
N1	0.2385 (7)	0.8835 (5)	1.0100 (6)	0.0247 (16)	
N2	0.2537 (7)	1.0444 (5)	0.9095 (6)	0.0252 (16)	
N3	0.3418 (6)	1.0118 (5)	0.7105 (5)	0.0227 (15)	
C1	0.1995 (7)	0.9617 (6)	1.0565 (6)	0.0210 (18)	
C2	0.1623 (9)	0.9559 (7)	1.1573 (7)	0.033 (2)	
H2	0.1349	1.0105	1.1889	0.040*	
C3	0.1660 (11)	0.8680 (8)	1.2116 (8)	0.044 (3)	
H3	0.1394	0.8630	1.2788	0.053*	
C4	0.2089 (10)	0.7900 (8)	1.1649 (8)	0.042 (3)	
H4	0.2155	0.7307	1.2006	0.051*	
C5	0.2422 (10)	0.8003 (7)	1.0642 (8)	0.036 (2)	
H5	0.2690	0.7460	1.0314	0.043*	
C6	0.2071 (8)	1.0522 (6)	0.9937 (7)	0.0251 (19)	
C7	0.1620 (9)	1.1447 (6)	1.0362 (8)	0.036 (2)	
H7A	0.2439	1.1770	1.0840	0.054*	
H7B	0.0967	1.1315	1.0797	0.054*	
H7C	0.1167	1.1847	0.9721	0.054*	
C8	0.2824 (10)	1.1260 (6)	0.8452 (8)	0.036 (2)	
H8A	0.3204	1.1785	0.8969	0.043*	
H8B	0.1954	1.1472	0.7902	0.043*	
C9	0.3870 (10)	1.0959 (6)	0.7849 (8)	0.037 (2)	
H9A	0.4031	1.1489	0.7388	0.045*	
H9B	0.4764	1.0815	0.8414	0.045*	
C10	0.4615 (9)	0.9792 (7)	0.6732 (8)	0.038 (2)	
H10A	0.5339	0.9538	0.7384	0.045*	
H10B	0.5020	1.0331	0.6434	0.045*	
C11	0.4195 (12)	0.9037 (7)	0.5826 (9)	0.046 (3)	
H11A	0.5026	0.8835	0.5610	0.055*	
H11B	0.3829	0.8487	0.6135	0.055*	
C12	0.1956 (11)	0.9629 (8)	0.5184 (8)	0.048 (3)	
H12A	0.1594	0.9073	0.5485	0.057*	
H12B	0.1223	0.9850	0.4516	0.057*	
C13	0.2283 (9)	1.0409 (7)	0.6081 (8)	0.036 (2)	
H13A	0.2573	1.0982	0.5759	0.043*	
H13B	0.1432	1.0558	0.6298	0.043*	

### Atomic displacement parameters (Å^2^)

**Table:**

	*U*^11^	*U*^22^	*U*^33^	*U*^12^	*U*^13^	*U*^23^
Zn1	0.0158 (5)	0.0246 (5)	0.0212 (5)	−0.0006 (4)	0.0046 (4)	−0.0044 (4)
Br1	0.0322 (6)	0.0540 (7)	0.0475 (7)	0.0094 (5)	0.0102 (5)	0.0007 (5)
Br2	0.0264 (5)	0.0500 (6)	0.0486 (7)	−0.0049 (4)	0.0101 (4)	−0.0081 (5)
O1	0.038 (4)	0.083 (5)	0.030 (4)	0.004 (4)	0.016 (3)	−0.002 (4)
N1	0.022 (4)	0.032 (4)	0.019 (4)	0.004 (3)	0.004 (3)	−0.001 (3)
N2	0.024 (4)	0.027 (4)	0.022 (4)	0.000 (3)	0.001 (3)	−0.004 (3)
N3	0.018 (3)	0.028 (4)	0.020 (4)	−0.003 (3)	0.004 (3)	0.005 (3)
C1	0.007 (4)	0.037 (5)	0.015 (4)	0.000 (3)	−0.003 (3)	−0.005 (3)
C2	0.025 (5)	0.049 (6)	0.027 (5)	−0.005 (4)	0.007 (4)	−0.015 (4)
C3	0.046 (6)	0.070 (8)	0.014 (5)	−0.009 (5)	0.006 (4)	0.002 (5)
C4	0.052 (6)	0.049 (6)	0.025 (5)	−0.006 (5)	0.010 (5)	0.009 (5)
C5	0.035 (5)	0.034 (5)	0.036 (6)	0.006 (4)	0.007 (4)	0.000 (4)
C6	0.021 (4)	0.028 (4)	0.023 (5)	−0.003 (3)	0.002 (4)	−0.010 (4)
C7	0.028 (5)	0.034 (5)	0.047 (6)	0.010 (4)	0.014 (4)	−0.012 (4)
C8	0.051 (6)	0.026 (5)	0.031 (5)	−0.008 (4)	0.012 (5)	−0.006 (4)
C9	0.040 (6)	0.032 (5)	0.037 (6)	−0.023 (4)	0.006 (4)	−0.002 (4)
C10	0.020 (5)	0.055 (6)	0.041 (6)	−0.003 (4)	0.011 (4)	0.008 (5)
C11	0.051 (6)	0.055 (7)	0.041 (6)	0.007 (5)	0.027 (5)	−0.003 (5)
C12	0.043 (6)	0.072 (8)	0.023 (5)	−0.005 (5)	0.002 (5)	−0.003 (5)
C13	0.030 (5)	0.042 (6)	0.035 (5)	0.002 (4)	0.007 (4)	0.008 (4)

### Geometric parameters (Å, °)

**Table:**

Zn1—N2	2.083 (7)	C4—H4	0.9300
Zn1—N1	2.235 (7)	C5—H5	0.9300
Zn1—N3	2.347 (6)	C6—C7	1.507 (11)
Zn1—Br1	2.3701 (15)	C7—H7A	0.9600
Zn1—Br2	2.3775 (15)	C7—H7B	0.9600
O1—C11	1.398 (12)	C7—H7C	0.9600
O1—C12	1.407 (12)	C8—C9	1.485 (13)
N1—C5	1.334 (11)	C8—H8A	0.9700
N1—C1	1.337 (10)	C8—H8B	0.9700
N2—C6	1.241 (11)	C9—H9A	0.9700
N2—C8	1.457 (11)	C9—H9B	0.9700
N3—C10	1.451 (11)	C10—C11	1.496 (13)
N3—C13	1.474 (10)	C10—H10A	0.9700
N3—C9	1.474 (10)	C10—H10B	0.9700
C1—C2	1.380 (12)	C11—H11A	0.9700
C1—C6	1.492 (11)	C11—H11B	0.9700
C2—C3	1.391 (14)	C12—C13	1.512 (13)
C2—H2	0.9300	C12—H12A	0.9700
C3—C4	1.352 (14)	C12—H12B	0.9700
C3—H3	0.9300	C13—H13A	0.9700
C4—C5	1.365 (13)	C13—H13B	0.9700
			
N2—Zn1—N1	73.5 (3)	C6—C7—H7B	109.5
N2—Zn1—N3	79.4 (3)	H7A—C7—H7B	109.5
N1—Zn1—N3	151.0 (2)	C6—C7—H7C	109.5
N2—Zn1—Br1	133.48 (18)	H7A—C7—H7C	109.5
N1—Zn1—Br1	92.70 (17)	H7B—C7—H7C	109.5
N3—Zn1—Br1	98.80 (16)	N2—C8—C9	108.2 (7)
N2—Zn1—Br2	108.40 (19)	N2—C8—H8A	110.1
N1—Zn1—Br2	95.80 (18)	C9—C8—H8A	110.1
N3—Zn1—Br2	102.32 (16)	N2—C8—H8B	110.1
Br1—Zn1—Br2	117.20 (6)	C9—C8—H8B	110.1
C11—O1—C12	108.1 (7)	H8A—C8—H8B	108.4
C5—N1—C1	118.3 (8)	N3—C9—C8	113.6 (7)
C5—N1—Zn1	128.4 (6)	N3—C9—H9A	108.8
C1—N1—Zn1	113.1 (5)	C8—C9—H9A	108.8
C6—N2—C8	123.3 (7)	N3—C9—H9B	108.8
C6—N2—Zn1	121.2 (6)	C8—C9—H9B	108.8
C8—N2—Zn1	115.2 (5)	H9A—C9—H9B	107.7
C10—N3—C13	107.9 (7)	N3—C10—C11	112.0 (7)
C10—N3—C9	108.4 (7)	N3—C10—H10A	109.2
C13—N3—C9	108.8 (7)	C11—C10—H10A	109.2
C10—N3—Zn1	115.9 (5)	N3—C10—H10B	109.2
C13—N3—Zn1	115.9 (5)	C11—C10—H10B	109.2
C9—N3—Zn1	99.3 (5)	H10A—C10—H10B	107.9
N1—C1—C2	120.6 (8)	O1—C11—C10	112.0 (8)
N1—C1—C6	114.4 (7)	O1—C11—H11A	109.2
C2—C1—C6	124.9 (8)	C10—C11—H11A	109.2
C1—C2—C3	119.9 (9)	O1—C11—H11B	109.2
C1—C2—H2	120.1	C10—C11—H11B	109.2
C3—C2—H2	120.1	H11A—C11—H11B	107.9
C4—C3—C2	118.7 (9)	O1—C12—C13	112.0 (8)
C4—C3—H3	120.6	O1—C12—H12A	109.2
C2—C3—H3	120.6	C13—C12—H12A	109.2
C3—C4—C5	118.5 (9)	O1—C12—H12B	109.2
C3—C4—H4	120.7	C13—C12—H12B	109.2
C5—C4—H4	120.7	H12A—C12—H12B	107.9
N1—C5—C4	123.9 (9)	N3—C13—C12	111.5 (8)
N1—C5—H5	118.1	N3—C13—H13A	109.3
C4—C5—H5	118.1	C12—C13—H13A	109.3
N2—C6—C1	115.7 (7)	N3—C13—H13B	109.3
N2—C6—C7	125.1 (8)	C12—C13—H13B	109.3
C1—C6—C7	119.3 (8)	H13A—C13—H13B	108.0
C6—C7—H7A	109.5		
